# Magnified Dermoscopy in Skin Cancer and Infectious Skin Diseases

**DOI:** 10.3390/medicina61111970

**Published:** 2025-11-03

**Authors:** Katarzyna Korecka, Joanna Pogorzelska-Dyrbuś, Adriana Polańska, Aleksandra Dańczak-Pazdrowska, Aimilios Lallas

**Affiliations:** 1 Department of Dermatology, Poznań University of Medical Sciences, 61-701 Poznań, Poland; apolanska@ump.edu.pl (A.P.); aleksandra.danczak-pazdrowska@ump.edu.pl (A.D.-P.); 2 “Estevita” Specialist Medical Practice, 43-100 Tychy, Poland; jpogorzelskadyrbus@gmail.com; 3 First Department of Dermatology, School of Medicine, Faculty of Health Sciences, Aristotle University, GR-54124 Thessaloniki, Greece

**Keywords:** dermoscopy, high magnification dermoscopy, super high magnification dermoscopy

## Abstract

*Background and Objectives*: Dermoscopy is a non-invasive clinical tool that allows for the in vivo visualization of pigmented and non-pigmented structures in the epidermis and the papillary dermis. The standard handheld dermoscopy offers a magnification of 10×, whereas the videodermatoscopes can obtain a magnification of up to 140×. Recently, a new method called magnified dermoscopy was introduced, in which a magnification of 400× can be achieved. *Materials and Methods*: This review was conducted according to the Preferred Reporting Items for Systematic Reviews and Meta-Analyses (PRISMA) reporting guidelines. Comprehensive research was conducted using the PubMed database on 9 June 2025, using the following keywords: “high magnification” or “super high magnification” or “optical super high magnification” or “400×”, and “dermoscopy” or “dermatoscopy”. *Results*: From a total of 237 records retrieved, 25 were found to be suitable for this review, and consisted of: four prospective studies, three retrospective studies, six case series, ten case reports and two image letters. *Conclusions*: This review summarizes the current knowledge on magnified dermoscopy, compiling existing data and exploring future perspectives for this emerging non-invasive diagnostic method.

## 1. Introduction

Dermoscopy is a non-invasive clinical tool that allows the in vivo visualization of pigmented and non-pigmented structures located in the epidermis and the papillary dermis [1]. Its applications are wide, as it is used to evaluate suspicious lesions to exclude a potential melanoma [2], non-melanoma skin cancers [3] and premalignant lesions [4]. Additionally, it is frequently utilized as a supplementary method for diagnosing inflammatory conditions [5], hair disorders [6] and nail abnormalities [7]. The latest developments in dermoscopy allow for the examination of skin lesions with polarized and non-polarized light [8], as well as ultraviolet (UV) light [9]. Handheld dermatoscopes offer a magnification of ×10, while videodermatoscopes can achieve magnification levels of up to 140×.

The initial observations of melanocytic lesions examined at 400× magnification were published in 1993 by Puppin et al. [10]. The researchers used a fiber-optic camera with various lenses that permitted magnification ranging from 10× to 400×, which was directly connected with a digital camera for image capturing. In 2018, Dusi et al. used a 400× lens from the Fotofinder System (Bad Birnbach, Germany) [11] to capture images of junctional and dermal nevus cells, indicating the onset of a new era in dermoscopy [12]. Subsequently, in 2019, Cinotti et al. [12] correlated the images of solar lentigo and melanoma structures observed under 400× dermoscopy to those obtained via reflectance confocal microscopy (RCM).

Currently, there are only a limited number of devices that provide magnification levels of up to 400×. The most extensively researched devices are the Fotofinder, which offers a field of view (FOV) of 1 mm × 0.5625 mm, and a Horus device (Horus system, Trapani, Italy) with a FOV of 1.7 mm × 1.3 mm) [13]. In the available literature, the analysis of lesions at 400× magnification has been termed optical super high magnification dermoscopy (OSHMD) [13]. However, this term has been recently replaced by “magnified dermoscopy” (MD).

This review summarizes the current knowledge on magnified dermoscopy, compiling existing data and exploring future perspectives for this emerging non-invasive diagnostic method.

## 2. Materials and Methods

This review was conducted according to the Preferred Reporting Items for Systematic Reviews and Meta-Analyses (PRISMA) reporting guidelines. Comprehensive research was conducted using the PubMed database on 9 June 2025, using the following keywords: “high magnification” or “super high magnification” or “optical super high magnification” or “400×”, and “dermoscopy” or “dermatoscopy”. Two independent researchers reviewed all available records to ensure accuracy. Excluded records included those unrelated to the topic, reviews, short communications, non-English manuscripts, studies on fluorescence advanced videodermoscopy (considered a separate topic with limited research) and studies using a magnification below 400×. Each study was assigned a level of evidence according to the 2011 Oxford Centre for Evidence-Based Medicine levels of evidence guidelines.

**Figure 1:**
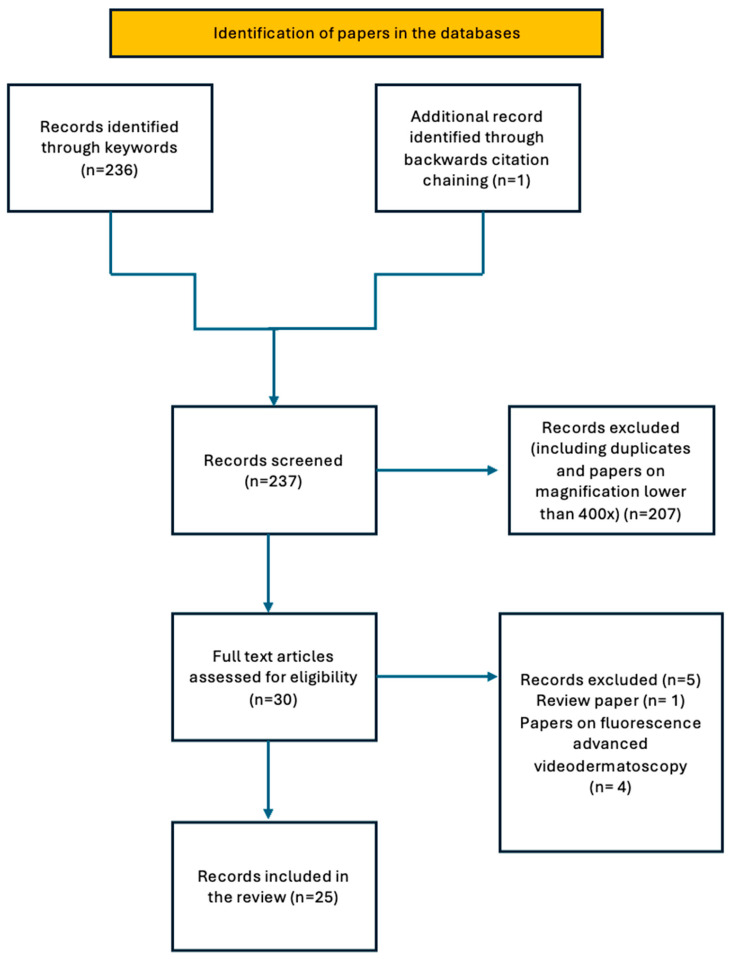
A diagram showing the study process and the data selection steps.

From a total of 237 records retrieved, 25 were found to be suitable for this review, and consisted of: 4 prospective studies, 3 retrospective studies, 6 case series, 10 case reports and 2 image letters (Figure 1).

The summary of the assessed research is available in Table 1.

## 3. Results

### 3.1. Magnified Dermoscopy in Melanocytic Lesions

Pigmented melanocytic lesions are the most extensively studied group under magnified dermoscopy. Since early melanomas and atypical nevi may exhibit similar characteristics, new applications of dermoscopy are currently being investigated to avoid unnecessary excisions.

**Figure 2:**
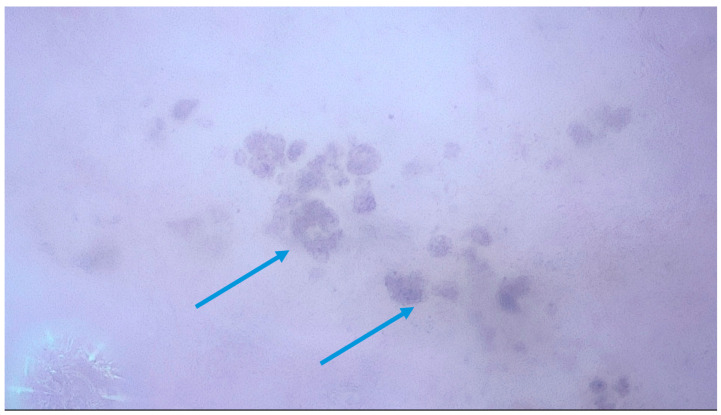
Heterogenous, blue-purple structures (blue arrow), corresponding to nesting melanocytes in an invasive melanoma (400× magnification).

In a multicentric observational study by Cinotti et al. [14], 79 patients with 51 nevi and 31 melanomas were examined with magnified dermoscopy. In melanomas, pigmented cells that were larger than keratinocytes (87.1%), irregular in size and shape (74.2%), out of focus (51.6%) and violet/blue in color (61.3%) were the most frequently observed features (Figure 2). A network without edge papillae (64.5%), melanophages (48.4%), violet/blue nests (29%) and out-of-focus blue structureless areas (61.3%) prevailed in melanoma. In contrast, the cells observed in nevi were more polygonal (91.2%), in focus (89.5%) and were distributed in nests inside the network or outside dermal papillae 56.1%). Another study by Cinotti et al. [13] on 190 pigmented, non-facial lesions included 73 melanomas and 117 benign lesions. Roundish (49.3%), dendritic (30.1%), irregularly arranged (41.1%) and irregular in shape and size (52.1%) melanocytes and angled nests (22.2%) were more frequent in melanomas. Benign lesions displayed mainly keratinocytes (93.2%), roundish nests (35.9%) and, less frequently, melanophages (16.2%).

In 2023, a case report on melanoma cells in magnified dermoscopy and their correspondence with histopathology was published by Pogorzelska-Dyrbuś et al. [19]. The presence of scattered irregular round pigmented cells of different sizes and a nest of pigmented cells which corresponded to pagetoid spread on pathology was reported, in line with a previous study [12].

In a retrospective observational study by Winkler et al. [15] on 99 patients with 65 nevi, 11 melanomas in situ, 22 invasive melanomas and one melanoma metastasis, the findings of the magnified dermoscopy were correlated with histopathological images. In melanomas, irregular distribution of cells along the pigment network (76.5%), different sizes of cells (79.4%), irregular vessels (32.4%), red (64.7%) and blue (55.9%) structureless areas and black-grey dots (47.1%) were found. In nevi, a regular network (61.5%) and regular distribution of cells along the pigment network (60%) with the same size (72.3%) were described as the most prominent features. Comparing the magnified dermoscopy images with pathological slides allowed the authors to correlate polygonal brown cells inside the network to keratinocytes, large roundish cells in the irregular distribution of atypical melanocytes and grey out-of-focus cells to melanophages. Therefore, grey out-of-focus cells were found in regression. Moreover, black dots corresponded to single melanocytes within the epidermis, greyish pigmentation next to dots to confluent tumor nests in the dermis and small roundish nests were consistent with aggregated tumor cells in melanomas. The authors hypothesize that the color of the nests could suggest the location of the tumor within the epidermis or dermis. Blue areas corresponded to a deeply distributed pigment, which is often also visible in classic dermoscopy of blue nevi or invasive melanomas.

Lentigo maligna (LM) remains a difficult entity to diagnose, although different algorithms have been proposed so far in standard dermoscopy, especially on the face [37]. These lesions have also been studied in videodermoscopy and fluorescence advanced videodermoscopy [38]. In a retrospective, observational, multicentric study by Cinotti et al. [16], a group of 61 patients with 23 LM, 3 LM melanomas (LMM), 15 solar lentigines, 12 seborrheic keratoses, six lichenoid keratoses and two pigmented actinic keratoses were examined. The most common features in the LM/LMM group were dendritic melanocytes (96.2%), roundish melanocytes (92.3%), folliculotropism of melanocytes (92.3%), melanocytes with irregular arrangement (76.9%) and irregularity of melanocytes in shape and size (50%). The authors suggested that combining standard dermoscopy with magnified dermoscopy, particularly for detecting folliculotropism and identifying atypical melanocytic cells, could improve the recognition of atypical melanocytic lesions. Additionally, magnified dermoscopy may be useful in selecting the optimal site for a diagnostic biopsy.

Genital pigmented lesions represent another diagnostically challenging category, with limited available data [39]. A study by Ravni et al. [17] analyzed 207 lesions in 152 patients, with 32 pathologically confirmed tumors (one BCC, two condylomas, nineteen melanoses, two melanomas in situ, eight nevi), and 164 melanosis and 11 nevi assessed with magnified dermoscopy. The authors compared the available magnified dermoscopy data with RCM attributes. In melanosis, ring patterns and isolated round cells were observed at a roughly similar frequency between confocal microscopy and magnified dermoscopy. Moreover, dendritic cells and round cells in nests were less frequently observed with magnified dermoscopy than with confocal microscopy.

Some published reports have also described single cases of halo nevus, Spitz nevus and atypical Spitz tumors, but these descriptions require further elaboration [20,21,22].

A case series correlating histopathology features with the magnified dermoscopy features of junctional, dermal and compound nevi was published by Pogorzelska-Dyrbuś et al. [23]. In junctional nevi, numerous pigmented polygonal and roundish structures corresponding to keratinocytes and melanocytes were detected. Purple structures, larger than keratinocytes and normal melanocytes, were correlated with diffusely filled melanin melanophages. The magnified dermoscopy pattern of compound nevi was similar to junctional nevi. Furthermore, the authors of this article emphasize that atypical melanocytes in both atypical nevi and melanoma are larger and heterogeneous in shape and size. In dermal nevi, there are numerous brown, roundish structures with a darker rim and an internal lighter part. In addition, in darker ones, purple multi-shaped larger pigmented structures were observed. Roundish structures histologically corresponded to melanocytes, while multi-shaped structures corresponded to melanophages. Similarly, in a case report of dermal nevus on a genital region in a 20-year-old patient, circular melanocytes that were regular and uniform in size were described [18].

**Figure 3:**
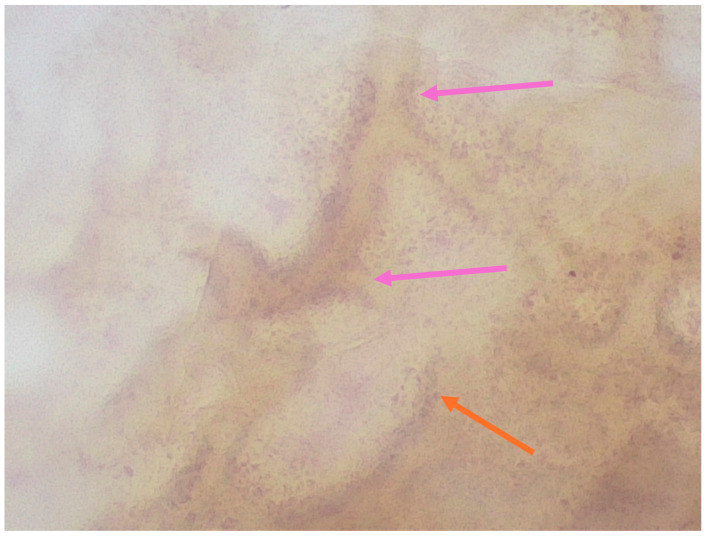
Nevus with network (marked with pink arrows), with polygonal structures corresponding to pigmented keratinocytes (orange arrows) (400× magnification).

Exemplary images of a melanoma and nevus in magnified dermoscopy are presented in Figure 2 and Figure 3.

### 3.2. Magnified Dermoscopy in Solar Lentigo and Lichen Planus-like Keratosis

The magnified dermoscopy features of solar lentigo and lichen planus-like keratosis (lplk) were described in a case report by Pogorzelska-Dyrbuś et al. [24]. The authors describe a 73-year-old patient with a solar lentigo on the cheek, in which magnified dermoscopy revealed clearly visible brown-reddish uniform polygonal structures, corresponding to keratinocytes that contoured follicular openings. Furthermore, areas of dense arrangements of brown polygonal structures with well-defined borders were reported.

In a 55-year-old patient with a grey macule of the arm, histopathologically confirmed as lplk, numerous blue-purple large structures corresponding to melanophages with straight linear vessels were described [24].

### 3.3. Magnified Dermoscopy in Basal Cell Carcinoma

The only available prospective observational study was published by Pogorzelska-Dyrbuś et al. [25], and featured 41 BCCs: nodular (61%), superficial (26.8%), multifocal (9.8%) and infiltrative (2.4%). The authors compared the frequency of the examined features between magnified dermoscopy and conventional dermoscopy. The percentage of looped vessels was significantly higher in magnified dermoscopy than in conventional dermoscopy (63.4% vs. 29.2%). Arborizing vessels were seen at the same frequency in both magnifications (53.7%). Pigmented structures were more common in magnified dermoscopy than in conventional dermoscopy (56.1% vs. 34.1%), with the identification of individual cells, including melanophages.

Magnified dermoscopy might be helpful in differentiating BCCs from dermal nevi. A study by Pogorzelska-Dyrbuś et al. [27] suggested that looped or branched vessels are present in both, while, in dermal nevi, roundish pigmented structures are observed, which most likely corresponded to melanocytes. In BCC, however, pigmented roundish structures were absent, but aggregated fine pigmented structures were observed. Therefore, it appears that magnified dermoscopy may allow for different diagnoses of these two skin lesions [26,28].

### 3.4. Magnified Dermoscopy in Infectious Diseases

Di Bartolomeo et al. [29] published a prospective study on the treatment follow-up for 22 patients with scabies observed under magnified dermoscopy. Intestinal peristalsis or movement of surface structures were suggested as signs of mite vitality, while a translucent appearance of “delta wing” (head and legs) or visualization of the epimeres of anterior legs at a higher magnification were associated with the degradation of the mite. A more detailed description of the visualization of scabies mite under magnified dermoscopy was provided in two case reports [30,31].

A case series by Di Bartolomeo et al. described the application of magnified dermoscopy in detecting *Pthirus pubis* and *Pediculus capitis* infection [32]. Furthermore, magnified dermoscopy has been used in one case report of tinea negra, where it showed brown elongated hyphae, filaments and two-celled spindle-shaped conidia [33]. *Demodex folliculorum* mites, which can be observed as white structures inside the hair follicle, have also been described to be visible in magnified dermoscopy [32,34]. A finding of *Neotrombicula autumnalis* in magnified dermoscopy was described by Orsini et al. [35], while 400× magnification was also helpful for detecting a tick bite, and to ensure the correct removal of the tick [36].

## 4. Discussion

Dermoscopy is a well-established, non-invasive diagnostic technique that is routinely used in clinical dermatology. Nevertheless, clinicians continue to explore novel modalities to enhance diagnostic precision. Reflectance confocal microscopy (RCM) and line-field confocal optical coherence tomography (LC-OCT) have gradually been integrated into clinical practice as new imaging tools. Magnified dermoscopy enables more detailed visualization of the tumors’ structures and may prove valuable in delineating surgical margins preoperatively, as it allows us to analyze the lesion on a cellular level. However, determining the proper shape of melanocytes or keratinocytes in order to set a proper diagnosis requires precision and experience.

Moreover, this method could potentially be applied for monitoring therapeutic responses in non-invasive therapeutic modalities. Regardless, these applications warrant further clinical investigation and more clinical studies, as the technique is still relatively new and might be difficult for an inexperienced clinician.

## 5. Current Limitations

**Figure 4:**
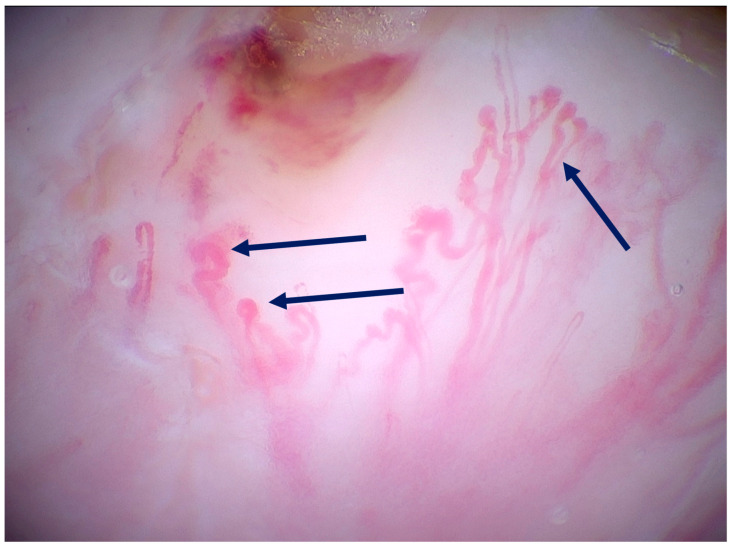
Looped vessels. The U-shape at the end of each vessel is marked with dark blue arrows (400× magnification).

**Figure 5:**
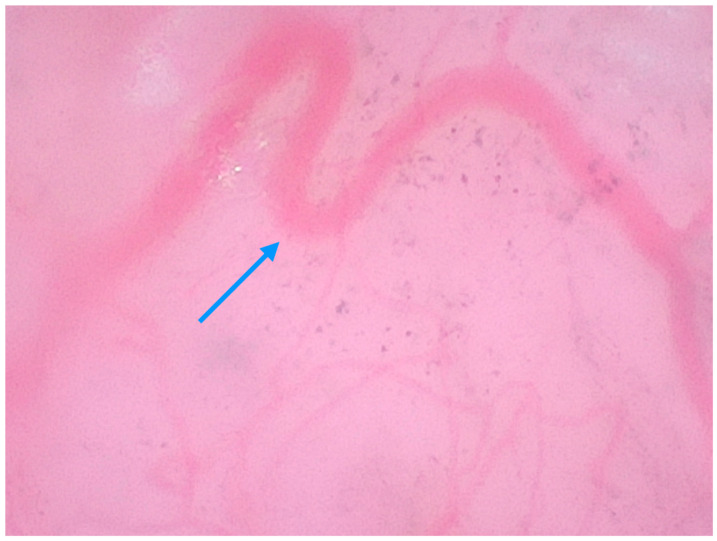
Arborizing vessel (arrow) in a BCC (400× magnification).

Since magnified dermoscopy is still a relatively new method, terminology is still being developed. A diverse group of specialists evaluated 74 nevi and 20 melanomas, but substantial agreement was only reached for vascular structures. Examples of vascular structures seen in magnified dermoscopy are presented in Figure 4 and Figure 5. Fair agreement was observed for cell irregularity and network characteristics, while overall agreement on other magnified dermoscopy features remained poor [40]. Recently, Guida et al. [41] published a consensus of the International Dermoscopy Society task force, in which the experts agreed on the proposed terminology concerning the main magnified dermoscopy features.

Additionally, data on magnified dermoscopy are limited, as the device is less accessible than a handheld dermoscope or videodermatoscopes. Given that magnified dermoscopy visualizes structures at the cellular level, image evaluation requires correlation with histopathology. However, there is still little research comparing magnified dermoscopy images with pathological slides.

A drawback of the available devices is their limited field of view. Since multiple photographs are often required, capturing high-resolution images can be time-consuming, making the process less practical in a clinical setting. Another limitation is the poor image acquisition in hyperkeratotic or heavily pigmented lesions [13].

Despite these challenges, magnified dermoscopy is a promising technique for an advanced morphologic assessment of skin diseases.

## 6. Conclusions

Magnified dermoscopy is an emerging and promising technique in dermatology. As a novel diagnostic tool, it offers opportunities to explore new applications, particularly in the fields of skin cancer detection and infectoscopy. However, further studies involving larger cohorts and correlations with histopathological findings are required to validate its clinical utility.

## Figures and Tables

**Table 1:** Summary of all the available data on magnified dermoscopy.

Author and Corresponding Reference	Study Type/Case Report/Case Series	Number of Lesions and Type of Lesions	Findings in 400× Dermoscopy	Oxford Level of Evidence Ox
**Melanocytic lesions**				
Cinotti et al. (2023) [13]	Retrospective, observational multicentric study (University of Sienna and Saint-Etienne, Department of Dermatology Senigallia, Private Practice in Genoa) excluding lesions on the face.	190 patients, 73 melanomas and 117 benign lesions (including 17 Reed/Spitz nevi)—total, 190 lesions.	**Melanomas**: Roundish (49.3%), dendritic (30.1%), irregularly arranged (41.1%), irregular in shape and size (52.1%) melanocytes, angled nests (22.2%). **Nevi**: Network with edged papillae (25.6%). **Benign lesions:** higher visibility of keratinocytes (93.2%), roundish nests (35.9%), lower presence of melanophages (16.2%)—no statistical relevance. Cell color (black 13 (11.1%) vs. 7 (9.6%), brown 107 (91.5%) vs. 66 (90.4%), violet/blue 45 (38.5%) vs. 29 (39.7%), out-of-focus bluish (36 (30.8%) vs. 24 (32.9%)) or grey/brown (23 (19.7) vs. 11 (15.1%)) structureless areas and vessel presence (26 (22.2%) vs. 23 (31.5%)) **were not statistically significant for the differential diagnosis between melanoma and benign lesions.**	III
Cinotti et al. (2021) [14]	Prospective, observational multicenter study (University of Siena and Saint-Etienne)	79 patients, 51 nevi and (including 7 Reed/Spitz Nevi and 2 blue nevi) and 31 melanomas (21 invasive melanomas and 4 lentigo maligna melanomas)—total, 88 lesions.	**Melanoma**: Pigmented cells larger than keratinocytes (87.1%), irregular in size and shape (74.2%), out of focus (51.6%), violet/blue in color (61.3%). Presence of melanophages (48.4%), violet/blue nests (29%), out of focus blue structureless areas (61.3%). Vessels: irregular in shape (32.3%) and dilated inside dermal papillae (19.4%) Network without edge papillae (64.5%). **Nevi:** Cells more polygonal (91.2%), in focus (89.5%) and distributed in nests inside the network or outside dermal papillae (56.1%), network with edged papillae (38.6%).	**III**
Winkler et al. (2025) [15]	Retrospective, observational study (University Hospital of Heidelberg)	99 patients, 65 nevi, 11 melanomas in situ, 22 invasive melanomas, 1 melanoma metastasis, total, 99 lesions.	**Melanomas**: In lesion parts (52.9%), irregular distribution of cells along pigment network (76.5%), different size of cells (79.4%), irregular vessels (32.4%), red (64.7%) and blue (55.9%) structureless areas, black-grey dots (47.1%). **Nevi**: Regular network (61.5%), regular distribution of cells along pigment network (60%) with the same size (72.3%)	III
Cinotti et al. (2023) [16]	Retrospective, observational multicentric study (University of Siena and Saint-Etienne) on facial lesions.	61 patients, lesions on the face: 23 lentigo maligna, 3 lentigo maligna melanoma, 15 solar lentigines, 12 seborrheic keratoses, 6 lichenoid keratoses, 2 pigmented actinic keratoses—total, 61 lesions.	**Lentigo maligna/lentigo maligna melanoma**: Dendritic melanocytes (96.2%), roundish melanocytes (92.3%), folliculotropism of melanocytes (92.3%), melanocytes with irregular arrangement (76.9%), irregularity of melanocytes in shapes and size (50%).	III
Ravni et al. (2024) [17]	Cross-sectional monocentric prospective study on genital, pigmented lesions (University Hospital of Saint-Etienne)	152 patients, 32 pathologically confirmed (1 BCC, 2 condylomas, 19 melanoses, 2 melanomas in situ, 8 nevi); no histology, only RCM and dermoscopy follow up: 164 melanoses, 11 nevi—total, 207 lesions.	**Melanosis**: Ring patterns and isolated round cells were observed roughly at a similar frequency between confocal microscopy and magnified dermoscopy. Dendritic cells and round cells in nests were less frequently observed with magnified dermoscopy than confocal microscopy. The concordance between magnified dermoscopy and confocal microscopy criteria for each evaluator was moderate to strong (kappa: 0.64, 0.53, 0.49) for the identification of the ring pattern, moderate to strong (kappa: 0.65, 0.75, 0.55) for the identification of the nests of round cells, strong (kappa: 0.63, 0.78, 0.76) for dendritic cells), strong (kappa: 0.73, 0.62, 0.63) for plump cellsand perfect (kappa: 1, 1, 1) for isolated round cells and spindle cells.	III
Cinotti et al. (2019) [12]	Image letter		**Solar lentigo in** magnified dermoscopy: Keratinocytes of solar lentigo appear as light brown polygons corresponding to hyper-reflective homogeneous cells in RCM. **Melanoma in** magnified dermoscopy: Pigmented roundish irregular structures which correlate with pagetoid spread in RCM.	V
Radi et al. (2022) [18]	Case report	20-year-old patient with genital lesion–dermal nevus.	**Dermal nevus findings**: Circular melanocytes, regular and uniform in size, some more pigmented than others, which appear as small circles, non-pigmented center surrounded by a peripheral pigmented ring, while others present full, solid small circles with a uniformly pigmented center and periphery.	V
Pogorzelska-Dyrbuś et al. (2023) [19]	Case report	38-year-old patient with a melanoma.	**Melanoma findings:** The presence of scattered irregular round pigmented cells of different sizes and a nest of pigmented cells which correspond to pagetoid spread on pathology.	V
Pogorzelska-Dyrbuś et. al. (2023) [20]	Case report	6-year-old patient with a halo nevus.	**Halo nevus findings**: Remnants of nests of melanocytes with a dilated vessel, the structure of a hair with completely depigmented cortex and white medulla at the periphery of the lesion along the normally pigmented hair.	V
Provvidenziale et al. (2020) [21]	Case report	11-year-old boy with a Spitz nevus on the right forearm.	**Spitz nevus findings:** Irregular in shape and size, dark brown and black pigmented cells, regular light brown pigmented cells. In pathology, they might correspond to melanocyte proliferation and pigmented keratinocytes.	V
Daviti et al. (2024) [22]	Case report	64-year-old patient with an atypical Spitz tumor.	**Atypical Spitz tumor findings:** Scattered, irregular, pigmented roundish structures and roundish structures with projections that are heterogenous in size.	V
Pogorzelska-Dyrbuś et al. (2025) [23]	Case series	Comparison between magnified dermoscopy features in dermal nevus, junctional nevus, compound nevus, atypical nevus and melanoma.	**Dermal nevus**: Numerous brown roundish structures with a darker rim and an internal lighter part, in darker types, purple multi-shaped larger pigmented structures are observed, histopathologically corresponding to melanophages. **Junctional nevus:** Polygonal and roundish structures histopathologically corresponding to either keratinocytes or melanocytes, which are difficult to distinguish with magnified dermoscopy. **Compound nevus**: Numerous pigmented cells are visible, the majority most probably corresponding to keratinocytes. **Atypical nevus**: Melanocytes are larger, either round or spindle-shaped. **Melanoma**: Melanocytes in melanoma are larger and heterogeneous in shape and size, unevenly scattered.	IV
**Solar lentigo and lichen planus-like keratosis**				
Pogorzelska-Dyrbuś et al. (2025) [24]	Case series	A 73-year-old female patient with a solar lentigo on her left cheek, and a 55-year-old female patient with lplk on her right arm.	**Solar lentigo:** Brown-reddish uniform polygonal structures corresponding to keratinocytes that contoured follicular openings, areas of dense arrangement of brown polygonal structures with well-defined borders. **Lichen planus-like keratosis:** Numerous blue-purple large structures corresponding to melanophages with straight linear vessels.	V
**Basal Cell Carcinoma (BCC)**				
Pogorzelska-Dyrbuś et al. (2023) [25]	Prospective, observational study in a private practice clinic (Tychy).	41 patients, 41 BCCs: nodular (61%), superficial (26.8%), multifocal (9.8%), infiltrative (2.4%).	**BCC findings:** The percentage of looped vessels was significantly higher in magnified dermoscopy than in standard dermoscopy (63.4% vs. 29.2%). Arborizing vessels were seen at the same frequency in both magnifications (53.7%). Pigmented structures were more common in magnified dermoscopy than in standard dermoscopy (56.1% vs. 34.1%), with the identification of individual cells, including melanophages.	III
Pogorzelska-Dyrbuś et al. (2022) [26]	Case series	2 cases of pigmented BCC (skin of abdomen in a 68-year-old male and left cheek in a 57-year-old female), 2 cases of nonpigmented BCCs (42-year-old female with a lesion on the back and 68-year-old male with a lesion on the forearm).	**Pigmented BCCs:** Tree-like vessels and linear vessels, arranged around blue-grey globules formed by nests of tumor cells in both cases. **Non-pigmented BCC:** Shiny white-red structureless area with looped vessels, with extremely branched loop seen in both cases.	V
Pogorzelska Dyrbuś et al. (2025) [27]	Case series	2 cases of dermal nevi (abdomen of a 36-year-old male, face 45-year-old female) and 2 cases of nodular BCCs (55-year-old and 45-year-old females).	**Dermal nevi:** Prominent stem and looped vessels, but also multiple small pale brown circular structures of the same size, possibly corresponding to melanocytes in the upper part of dermal nests. **BCCs:** Branched vessels and brown globules located focally at the periphery, fine pigmented structures and looped vessels.	
Pogorzelska-Dyrbuś et al. (2022) [28]	Case series	2 cases of BCC: nodular (back of an 81-year-old male patient) and superficial (abdomen of a 49-year-old male patient).	**Nodular BCC:** Looped vessels with extremely branched loops resembling oak leaves, which were distributed throughout the entire lesion. **Superficial BCC:** Numerous loop vessels forming the so-called “oak leaves”, distributed across the entire lesion.	V
**Infectoscopy**				
**Scabies**				
Di Bartolomeo et al. (2023) [29]	Prospective observational study (University of Messina, Italy)	22 patients were observed to evaluate treatment efficacy of benzyl benzoate 25% cream after failed 5% permetrine treatment.	Magnified dermoscopy **findings:** Intestinal peristalsis or movement of surface structures were suggested as signs of mite vitality, while translucent appearance of “delta wing” (head and legs) or visualization of the epimeres of anterior legs at higher magnification were associated with degradation of the mite.	IV
Winkler et al. (2022) [30]	Case report	75-year-old patient with treatment resistance lesions on upper trunk and axillary folds.	Magnified dermoscopy **findings:** Head, extremities and intestine of the mite more visible in classic dermoscopy.	V
Giuffrida et al. (2025) [31]	Image letter	Observation of scabies mites in magnified dermoscopy.	Magnified dermoscopy **findings**: Revealed details of head, extremities and intestine of the mite and larvae within eggs as sign of active infestation, invisible at lower magnifications, substantially obviating the need for mineral oil preparation.	V
**Other infectious diseases**				
Di Bartolomeo et al. (2024) [32]	Case series	Three patients with history of itch (50-year-old male, 2-year-old child, 40-year-old male) who were diagnosed with **Pthirus pubis, Pediculus capitis**, **scabies and demodex spp.**	Magnified dermoscopy **findings:** mouthparts with antennae, the digestive system, spiracles, terminal claws, hair on the dorsal surface, air tubes seen in Pthirus pubis specimen. In Pediculus capitis: antennae; mouthparts; eyes; digestive tract, legs were seen along vital nit, presenting the dome-shaped operculum. In third patient, scabiei mite with mouthparts and anterior legs, spines on abdomen and posterior legs visible through transparent body were detected, along with Demodex folliculorum tails.	V
Cinotti et al. (2019) [33]	Case Report	45-year-old male patient with a lesion on the palm, diagnosed as tinea egra caused by ***Hortaea werneckii***.	Magnified dermoscopy **findings:** brown elongated hyphae and two-celled spindle-shaped blastoconidia.	V
Cinotti et al. (2022) [34]	Case report	55-year-old male patient with papules on the face diagnosed as **demodicosis**.	Magnified dermoscopy **findings:** elongated and round white structures inside hair follicles which correspond to the image of Demodex folliculorum.	V
Orsini et al. (2023) [35]	Case report	27-year-old male patient with diffuse erythematous nodules, diagnosed as **trombiculosis**.	Magnified dermoscopy **findings:** six-legged golden colored parasites, strongly attached to the skin, which allowed us to diagnose an infestation of the larval stage of *Neotrombicula autumnalis*.	V
Di Bartolomeo et al. (2025) [36]	Case report	50-year-old female with a new lesion on her breast. After examination, a diagnosis of a **tick bite** was set.	Magnified dermoscopy **findings:** dorsal partial scutum of the tick, demonstrating that it was a female belonging to the Ixodidae family, and the intact rostrum, thus proving the correct removal of tick.	V

## Data Availability

No new data were created or analyzed in this study.

## References

[1] Campos-do-Carmo G., Ramos-e-Silva M. (2008). Dermoscopy: Basic Concepts. Int. J. Dermatol..

[2] Braun R.P., Rabinovitz H.S., Oliviero M., Kopf A.W., Saurat J.-H. (2005). Dermoscopy of Pigmented Skin Lesions. J. Am. Acad. Dermatol..

[3] Kato J., Horimoto K., Sato S., Minowa T., Uhara H. (2019). Dermoscopy of Melanoma and Non-Melanoma Skin Cancers. Front. Med..

[4] Zalaudek I., Argenziano G. (2015). Dermoscopy of Actinic Keratosis, Intraepidermal Carcinoma and Squamous Cell Carcinoma. Actinic Keratosis.

[5] Errichetti E. (2019). Dermoscopy of Inflammatory Dermatoses (Inflammoscopy): An Up-to-Date Overview. Dermatol. Pract. Concept..

[6] Rudnicka L., Olszewska M., Waśkiel A., Rakowska A. (2018). Trichoscopy in Hair Shaft Disorders. Dermatol. Clin..

[7] Thomas L., Dalle S. (2007). Dermoscopy Provides Useful Information for the Management of Melanonychia Striata. Dermatol. Ther..

[8] Benvenuto-Andrade C., Dusza S.W., Agero A.L.C., Scope A., Rajadhyaksha M., Halpern A.C., Marghoob A.A. (2007). Differences Between Polarized Light Dermoscopy and Immersion Contact Dermoscopy for the Evaluation of Skin Lesions. Arch. Dermatol..

[9] Pietkiewicz P., Navarrete-Dechent C., Togawa Y., Szlązak P., Salwowska N., Marghoob A.A., Leszczyńska-Pietkiewicz A., Errichetti E. (2024). Applications of Ultraviolet and Sub-Ultraviolet Dermatoscopy in Neoplastic and Non-Neoplastic Dermatoses: A Systematic Review. Dermatol. Ther..

[10] Puppin D., Salomon D., Saurat J.-H. (1993). Amplified Surface Microscopy. J. Am. Acad. Dermatol..

[11] Dusi D., Rossi R., Simonacci M., Ferrara G. (2018). Image Gallery: The New Age of Dermoscopy: Optical Super-High Magnification. Br. J. Dermatol..

[12] Cinotti E., Rossi R., Ferrara G., Tognetti L., Rubegni P., Perrot J.L. (2019). Image Gallery: Super-high Magnification Dermoscopy Can Identify Pigmented Cells: Correlation with Reflectance Confocal Microscopy. Br. J. Dermatol..

[13] Cinotti E., Cioppa V., Tognetti L., Perrot J.L., Rossi R., Gnone M., Cartocci A., Rubegni P., Cortonesi G. (2023). Super-High Magnification Dermoscopy in 190 Clinically Atypical Pigmented Lesions. Diagnostics.

[14] Cinotti E., Tognetti L., Campoli M., Liso F., Cicigoi A., Cartocci A., Rossi R., Rubegni P., Perrot J.L. (2021). Super-high Magnification Dermoscopy Can Aid the Differential Diagnosis between Melanoma and Atypical Naevi. Clin. Exp. Dermatol..

[15] Winkler J.K., Kommoss K.S., Vollmer A.S., Enk A.H., Haenssle H.A., Toberer F. (2025). Optical Super-high Magnification Dermoscopy of Benign and Malignant Melanocytic Lesions in Correlation with Histopathology. JDDG J. Dtsch. Dermatol. Ges..

[16] Cinotti E., Cartocci A., Liso F.G., Cioppa V., Falcinelli F., Tognetti L., Rubegni P., Perrot J.L. (2023). Super-High Magnification Dermoscopy Can Help for the Diagnosis of Lentigo Maligna: A Pilot Study on 61 Cases. Dermatol. Pract. Concept..

[17] Ravni E., Skowron F., David C., Cortez C.D., Lima S., Tognetti L., Chazelle M., Habougit C., Vercherin P., Perrot M. (2024). Correlation between Super-High Magnification (400x) Dermoscopy and Reflectance Confocal Microscopy for the Diagnosis of Melanosis and Other Pigmented Genital Lesions. Eur. J. Dermatol..

[18] Radi G., Rossi R., Diotallevi F., Giannoni M., Molinelli E., Paolinelli M., Ferrara G., Offidani A. (2023). The Role of the Optical Super High Magnification Dermoscopy (O.S.H.M.D) in the Management of Melanocytic Lesions. J. Eur. Acad. Dermatol. Venereol..

[19] Pogorzelska-Dyrbuś J., Szepietowski J.C. (2023). Melanoma Cells in Optical Super-High Magnification Dermoscopy. Br. J. Dermatol..

[20] Pogorzelska-Dyrbuś J., Cinotti E., Szepietowski J.C. (2023). Optical Super-High Magnification Dermoscopy Findings of Halo Naevus. Acta Derm. Venereol..

[21] Provvidenziale L., Cinotti E., Campoli M., Rubegni P. (2021). Superhigh Magnification Dermoscopy and Management of a Pediatric Spitz Nevus Mimicking Melanoma. Ital. J. Dermatol. Venereol..

[22] Daviti M., Papadimitriou I., Cinotti E., Lallas A. (2024). Atypical Melanocytes of an Atypical Spitz Tumour Observed with Optical Super-High Magnification Dermoscopy. Br. J. Dermatol..

[23] Pogorzelska-Dyrbuś J., Guida S., Radi G., Rossi R., Lallas A., Cinotti E. (2025). Correspondence of Optical Super-High Magnification Dermoscopy with Histopathology of Melanocytic Lesions. Dermatol. Pract. Concept..

[24] Pogorzelska-Dyrbuś J., Ławniczak-Cielińska D., Cinotti E. (2025). Optical Super-High Magnification Dermoscopy of Solar Lentigo and Lichen Planus-Like Keratosis. Dermatol. Pract. Concept..

[25] Pogorzelska-Dyrbuś J., Lallas A., Szepietowski J.C. (2023). Morphology of Vessels in Basal Cell Carcinoma in Optical Super-High Magnification Dermoscopy. Acta Derm. Venereol..

[26] Pogorzelska-Dyrbuś J., Szepietowski J.C. (2022). Optical Super-high Magnification Dermoscopy of Pigmented and Nonpigmented Nodular Basal Cell Carcinoma. J. Cosmet. Dermatol..

[27] Pogorzelska-Dyrbuś J., Cinotti E., Lallas A. (2024). Differentiation of Dermal Nevus and Basal Cell Carcinoma Based on Optical Super-High Magnification Dermoscopy. Dermatol. Pract. Concept..

[28] Pogorzelska-Dyrbuś J. (2022). “Oak-Leaf-like” Loop Vessels in Super-High Magnification Dermoscopy of Basal Cell Carcinoma. Dermatol. Pract. Concept..

[29] Di Bartolomeo L., Argenziano G., Borgia F., Vaccaro F., Vaccaro M. (2023). Efficacy Evaluation of Scabies Treatment through Super High Magnification Dermoscopy. Ital. J. Dermatol. Venereol..

[30] Winkler J.K., Toberer F., Enk A.H., Haenssle H.A. (2022). Super-high Magnification Dermatoscopy for In-vivo Imaging of Scabies Mites. JDDG J. Dtsch. Dermatol. Ges..

[31] Giuffrida R., Tognetti L., Guida S., Conforti C., Cinotti E., Zalaudek I., Guarneri F. (2025). High Magnification Dermoscopy for in Vivo Identification of Larvae within Eggs in Active Scabies Infestation. Br. J. Dermatol..

[32] Di Bartolomeo L., Vaccaro F., Borgia F., Macca L., Portuese S., Vaccaro M. (2024). Super-High Magnification Entodermoscopy: The New Era of Dermoscopy in the Field of Skin Parasitoses. Dermatol. Pract. Concept..

[33] Cinotti E., Ekinde S., Labeille B., Raberin H., Tognetti L., Rubegni P., Perrot J.L. (2019). Image Gallery: Pigmented Hyphae Can Be Identified in Vivo by High and Super-high Magnification Dermoscopy. Br. J. Dermatol..

[34] Cinotti E., Bertello M., Donelli C., Rossi R., Tognetti L., Perrot J.L., Rubegni P. (2023). Super-high Magnification Dermoscopy Can Detect *Demodex folliculorum*. J. Eur. Acad. Dermatol. Venereol..

[35] Orsini C., Cortonesi G., Lamberti A., Campoli M., Rubegni P., Cinotti E. (2023). Optical Super-High Magnification Dermoscopy: A Complementary Means in the Diagnosis of Trombiculosis. Dermatol. Pract. Concept..

[36] Di Bartolomeo L., Portuese S., Vaccaro F., Borgia F., Vaccaro M. (2024). Tick Bite and Super-High Magnification Dermoscopy. Dermatol. Pract. Concept..

[37] Micantonio T., Neri L., Longo C., Grassi S., Di Stefani A., Antonini A., Coco V., Fargnoli M.C., Argenziano G., Peris K. (2018). A New Dermoscopic Algorithm for the Differential Diagnosis of Facial Lentigo Maligna and Pigmented Actinic Keratosis. Eur. J. Dermatol..

[38] D’Onghia M., Falcinelli F., Barbarossa L., Pinto A., Cartocci A., Tognetti L., Rubegni G., Batsikosta A., Rubegni P., Cinotti E. (2025). Zoom-in Dermoscopy for Facial Tumors. Diagnostics.

[39] Lacarrubba F., Borghi A., Verzì A.E., Corazza M., Stinco G., Micali G. (2020). Dermoscopy of Genital Diseases: A Review. J. Eur. Acad. Dermatol. Venereol..

[40] Guida S., Kaleci S., Rossi R., Radi G., Molinelli E., Pellacani G., Cinotti E., Italian Optical Super-High Magnification Dermoscopy Group (2024). Optical Super-High Magnification Dermoscopy in the Diagnosis of Equivocal Melanocytic Lesions: Poor Agreement on Current Terminology and Future Perspectives. Dermatol. Pract. Concept..

[41] Guida S., Pogorzelska-Dyrbuś J., Radi G., Giuffrida R., Karls R., Daviti M., Rossi R., Hofmann-Wellenhof R., Perrot J., Garat H. (2025). Magnified Dermoscopy Terminology for Skin Tumours: International Dermoscopy Society Delphi Consensus. J. Eur. Acad. Dermatol. Venereol..

